# Comparison of early and intermediate-term outcomes between hybrid arch debranching and total arch replacement: A systematic review and meta-analysis of propensity-matched studies

**DOI:** 10.1371/journal.pone.0314341

**Published:** 2025-09-04

**Authors:** Naritsaret Kaewboonlert, Worawong Slisatkorn, Apichat Tantraworasin, Punthiti Pleehachinda, Tossapol Prapassaro, Natthipong Pongsuwan, Chanut Chatkaewpaisal, Tummarat Ruangpratyakul

**Affiliations:** 1 School of Surgery, Institute of Medicine, Suranaree University of Technology, Nakhon Ratchasima, Bangkok, Thailand; 2 Department of Surgery, Faculty of Medicine Siriraj Hospital, Mahidol University, Bangkok, Thailand; 3 Department of Surgery, Faculty of Medicine, Chiang Mai University, Chiang Mai, Thailand; 4 Department of Surgery, Phra Nakhon Si Ayutthaya Hospital, Phra Nakhon Si Ayutthaya, Thailand; 5 Heart Center, Bangkok Hospital Ratchasima, Nakhon Ratchasima, Bangkok, Thailand; 6 Department of Surgery, Roi Et Hospital, Roi Et, Thailand; Stanford University School of Medicine, UNITED STATES OF AMERICA

## Abstract

**Objectives:**

To systematically review propensity score-matched studies comparing hybrid arch repair (HAR) with total arch replacement (TAR) for aortic arch pathologies, summarizing early outcomes and intermediate-term results.

**Methods:**

We searched PubMed, Embase, the Cochrane Library, and Google Scholar to April 2024. The primary outcome was in-hospital mortality, evaluated by a random-effects model to calculate the odds ratio (OR). Time-to-event outcomes were synthesized as hazard ratios (HR) using inverse variance method.

**Results:**

Eight studies comprising 860 patients were included. There was no significant difference in in-hospital mortality between HAR and TAR groups (OR 0.66; 95% CI 0.33–1.31; p = 0.240). HAR was associated with a lower incidence of renal failure (OR 0.51; 95% CI 0.30–0.88; p = 0.020). In the isolated type A aortic dissection (ITAAD) subgroup, HAR showed a non-significant trend toward lower in-hospital mortality (OR 0.66; 95% CI 0.33–1.31, p = 0.24). In mixed degeneration-dissection (MDAD), TAR showed a non-significant trend toward lower risk of permanent neurological dysfunction (PND) (OR 2.84; 95% CI 0.89–9.10; p = 0.080) and a significantly lower three-year re-interventions rate (HR 2.99; 95% CI 1.48–6.04; p < 0.001). Other postoperative complications did not differ significantly: sternal re-entry for hemorrhage (OR 0.55; 95% CI 0.21–1.43; p = 0.220), and tracheostomy (OR 1.08; 95% CI 0.43–2.72; p = 0.870).

**Conclusions:**

HAR was associated with a lower risk of renal failure. In ITAAD, HAR showed a trend toward lower in-hospital mortality, whereas in MDAD cohorts, TAR showed a significantly lower three-year re-intervention rate. These findings should be interpreted with caution given the small number of studies and underlying heterogeneity. Further observational studies or randomized trials are warranted.

## Introduction

Atherosclerosis is the most common pathology of the aortic arch, followed by other conditions including aortic dissection [1,2]. Total arch replacement (TAR) is highly technically demanding, high-risk procedure for treating such pathologies [3,4]. In chronic aortic aneurysms, the goals of surgery are to prevent rupture or dissection and to minimize serious postoperative complications, including mortality, permanent neurological dysfunction, acute renal failure, and the need for tracheostomy. Moreover, the procedure indicated for the patients should significantly improve long-term survival probability and reduce the risk of disability [5,6].

Due to postoperative morbidity and mortality, less invasive therapeutic alternatives such as thoracic endovascular repair (TEVAR) combined with aortic arch surgical debranching or hybrid arch repair (HAR) [5] have become options for selected elderly patients or those unfit for conventional aortic repair (TAR).

In 2024, the guidelines from European Association for Cardio-Thoracic Surgery (EACTS) and the Society of Thoracic Surgeons (STS) [7] indicate emerging trend towards the utilization of TEVAR with HAR as an option for complicated aortic arch aneurysms or acute type A aortic dissections [8–10]. The advantages and drawbacks of HAR over TAR in the management of TAAD continue to be a subject of debate [10–12].

Currently, TAR is the standard of care for individuals with aortic arch aneurysms or TAAD. The individualized selection strategy for surgical treatment alternatives is controversial due to insufficient robust data comparing various strategies [7]. No randomized controlled trials have directly compared the outcomes of these treatment strategies. Most evidence on HAR versus TAR relies on observational studies.

Therefore, we conducted a meta-analysis to evaluate early clinical outcomes and intermediate-term survival of patients with aortic arch aneurysms or acute type A aortic dissection undergoing TAR or HAR. Included studies used propensity-score methods to mitigate selection bias.

## Materials and methods

This systematic review was conducted in accordance with the Preferred Reporting Items for Systematic Reviews and Meta-Analysis (PRISMA) guidelines established by Page et al. [13].

### Population, intervention, comparison, and outcome (PICO)

We employed the PICO framework, as delineated by Richardson et al. [14], the research questions was addressed as follows:

- **Population:** This includes any patients diagnosed with an aortic arch aneurysm or acute and chronic aortic dissections involving the aortic arch necessitating repair. The method used for repair technique was debranching combined with thoracic endovascular aortic repair. These patients were subsequently matched with similar characteristics to those who underwent conventional total arch replacement (in retrospective cohort studies utilizing propensity score matching).
- **Intervention:** All patients designated for hybrid arch debranching repair.
- **Comparison:** All patients designated for total arch replacement whose characteristics aligned with those of the intervention group.
- **Outcomes:** Outcome measurements were categorized into:
  - Early outcome measurements including in-hospital mortality, permanent neurological impairment or stroke, renal failure, sternal re-entry due to hemorrhage, and tracheostomy, were evaluated as odds ratio (OR).
  - Intermediate term outcome measures including 3-year survival rate and freedom from re-intervention were expressed as hazard ratio (HR)

### Search strategy

In April 2024, we conducted a search for studies relevant to this topic across multiple databases, including PubMed, Embase, Cochrane Library, and Google Scholar. We additionally conducted a manual search for references from published studies that fulfilled the criteria of this study. The inquiry included all domains under the headings: “hybrid arch” AND “total arch” AND “outcome” AND “aneurysm,” using medical subject headings (MeSH Terms). The Boolean operator ‘AND’ connected these concepts. The search was unrestricted by language or publication year. The titles of retrieved studies were initially examined, followed by a comprehensive assessment of the study abstracts and full texts to determine eligibility for inclusion.

### Eligible criteria

Studies were considered eligible if they fulfilled the following inclusion criteria: (1) prospective observational or retrospective cohort with a propensity-matched design, (2) comparison of outcomes in adults over 18 years of age, (3) reporting of early and intermediate outcomes. Studies were excluded if they encountered any of the following criteria: (1) participants were not propensity score matched, (2) case reports, correspondence, perspective pieces or review articles, (3) absence of aortic arch involvement, (4) extra-anatomical bypass or bypass technique not defined, and (5) involvement of thoracoabdominal aortic aneurysms.

### Data extraction and quality assessment

The literature search was conducted by two independent reviewers (NK and NP) using pre-established search strategies. Duplicate studies were systematically discarded. Each reviewer meticulously evaluated titles, abstracts, and, where necessary, full texts to identify studies that fulfilled the inclusion criteria. Data from the retrieved publications—including research information, design, patient demographics, treatment specifics, and early to intermediate outcomes--were extracted. The risk of bias was evaluated in accordance with the Cochrane Handbook for Systematic Reviews of Interventions [15], utilizing the Risk of Bias in Non-randomized Studies of Interventions (ROBINS-I) tool [16].

### Statistical analysis

The data was reported as medians with interquartile ranges using the approach of Hozo et al. [17]. Descriptive statistics were used to compare baseline characteristics between HAR and TAR, employing exact tests for categorical variables and independent samples t-tests for continuous variables, as appropriate. Early surgical outcomes were summarized as odds ratios (OR) for binary outcomes. Effect sizes for two-group comparisons were pooled with a random-effects model using restricted maximum-likelihood method. Survival and freedom from re-intervention were assessed with hazard ratios (HR). When HRs were not reported, data were extracted from Kaplan-Meier curves using WebPlotDigitizer (available from: https://automeris.io/WebPlotDigitizer). Subsequently, HRs with 95% confidence intervals (CI) were calculated from survival probabilities at discrete time points and the number of patients at-risk extracted from Kaplan-Meier curves. For pooling, HRs were natural logarithm transformed and combined by inverse variance method, subsequently re-expressed as the HR with 95%CI in the forest plot [18–20]. Subgroup meta-analyses were conducted to investigate the sources of heterogeneity, stratifying studies by population domain: isolated type A aortic dissections (ITAAD) versus mixed degeneration and dissection (MDAD) aortic arch pathology. Funnel plots symmetry was utilized to evaluate publication bias, and leave-one-out sensitivity analysis was employed to evaluate the robustness of the synthesized results. All tests were two-sided with p < 0.050 considered significant. Descriptive statistics, meta-analysis, and figure generation were performed in Stata 19 (Stata Corp, College Station, TX) and Microsoft Excel (Microsoft Corp., Redmond, WA).

## Results

### Literature search and study characteristics

**Fig 1:**
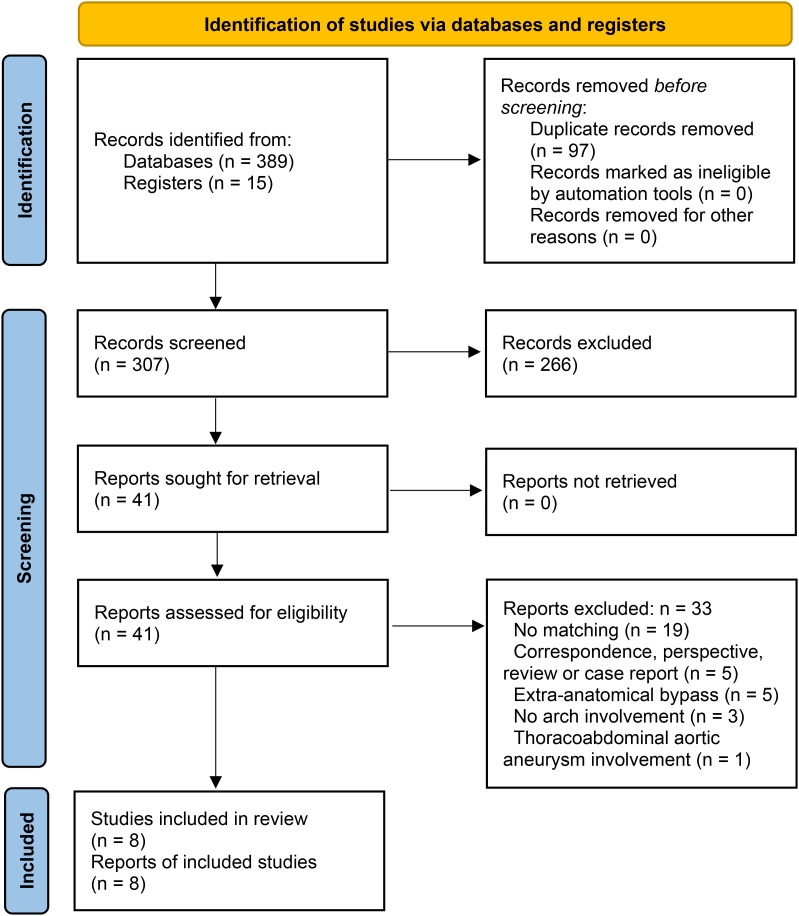
PRISMA flow diagram shows the systematic review process.

The process for selecting study is depicted in Fig 1. A total of 389 articles were identified, 97 records were discarded due to duplication, and 266 publications were excluded following screening. Additionally, 33 records were excluded for failing to meet the eligibility criteria: 19 were non-matching studies, 5 were correspondence, review articles, or case reports, 5 were extra-anatomical bypass or bypass technique not defined, 3 were not related to the aortic arch, and 1 involved a thoraco-abdominal aortic aneurysm. Table 1. outlines the study characteristics and the assessment of bias risks. All included studies adopt a retrospective cohort design and control for confounding factors utilizing propensity score matching. No studies describe the missing data in the cohort; this poses hazards for accurately reporting the effect magnitude of the missing data. Three of the eight studies [12,21–23] exhibited a significant risk of bias, as they neglected to incorporate well-known confounding factors, including cerebrovascular disease or chronic renal failure, in the propensity model (Table 1). One out of eight studies [23,24] exhibited a significant risk of bias due to deviations from intended interventions and the categorization of interventions. We evaluated four out of eight studies [12,21–28] as exhibiting a serious overall risk of bias, while the remaining studies [29–32] demonstrated a moderate overall risk of bias. All studies demonstrated a significant risk of bias (see supplementary information S1 Fig), highlighting the inherent limitations of non-randomized studies.

**Table 1:** Characteristics of studies included in this systematic review with risk of bias assessment.

Authors	Year	Country	Study size (n)	Pathology	ROBINS-I
Preventza O [29]	2015	United States	50	MDAD	Moderate
Tokuda Y [23]	2016	Japan	76	MDAD	Serious
Ma M [21]	2018	China	52	ITAAD	Serious
Joo HC [30]	2019	South Korea	96	MDAD	Moderate
Seike Y [12]	2019	Japan	100	MDAD	Serious
Liu Y [31]	2021	China	180	MDAD	Moderate
Huang F [22]	2022	China	70	ITAAD	Serious
Liu S [32]	2023	China	236	ITAAD	Moderate

Abbreviations: ROBINS-I, risk of bias in non-randomized studies: ITAAD, isolated type A aortic dissection: MDAD, mixed degeneration and dissection: TEVAR, thoracic endovascular aortic repair

### Patient characteristics

A total of 860 patients was enrolled in the studies, with 430 patients in each group, matched using the propensity score model (Table 2). The mean age for the HAR and TAR group was 68.3 ± 6.6 and 68.8 ± 6.3, respectively. No statistically significant difference was noticed between the mean ages of both groups (p = 0.256). The proportion of males in the HAR group (60.2%) and the TAR group (60.5%) exhibited no statistically significant difference (p = 1.000). The percentage of urgent and emergency operations was 33.0% in the HAR group and 35.3% in the TAR group, with no statistically significant difference (p = 0.637). No statistically significant difference was observed in the prevalence of underlying diseases, including hypertension, diabetes mellitus (DM), coronary artery disease (CAD), prior myocardial infarction (MI), chronic obstructive pulmonary disease (COPD), and renal failure.

**Table 2:** Summary of clinical characteristics reported in the included studies, presented as mean ± standard deviation or frequency and percentage.

Characteristics	Number of patients	HAR	Number of patients	TAR	p-value
Age, year	430	68.3 ± 6.6	430	68.8 ± 6.3	0.256
Male gender	430	259 (60.2)	430	260 (60.5)	1.000
Hypertension	342	241 (56.0)	342	243 (56.5)	0.953
Diabetes mellitus	342	39 (9.1)	342	39 (9.1)	1.000
Coronary artery disease or previous myocardial infarction	239	62 (14.4)	239	62 (14.4)	1.000
Chronic obstructive pulmonary disease	395	35 (8.1)	395	33 (7.7)	0.900
Renal failure	430	44 (10.2)	430	44 (10.2)	1.000
Urgent or emergency operation	430	142 (33.0)	430	152 (35.3)	0.637

Abbreviations: HAR, hybrid arch repair; TAR, total arch replacement.

### Early outcome

#### Early mortality analysis.

**Fig 2:**
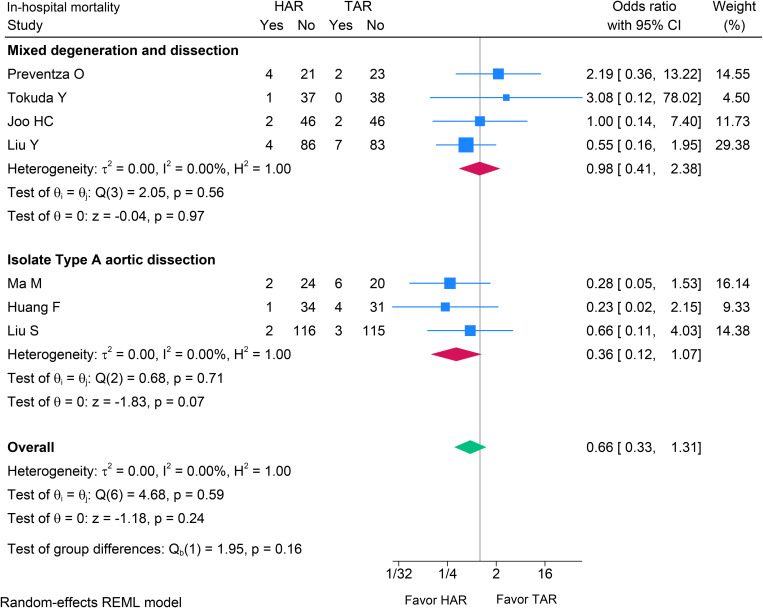
Forest plot showing the results of in-hospital mortality after HAR versus TAR with the subgroup meta-analysis in ITAAD and MDAD aortic arch pathologies. HAR: Hybrid Arch Repair; TAR: Total Arch Replacement; ITAAD: Isolated Type A Aortic Dissection; MDAD: Mixed Degeneration and Dissection.

All seven studies were included in a meta-analysis of in-hospital mortality. Forest plots were calculated and indicated an absence of heterogeneity (I^2^ = 0%). The comprehensive data indicated no significant difference in in-hospital mortality between the HAR and TAR groups (OR 0.66; 95% Cl 0.33–1.31; p = 0.240, I^2^ = 0%) (Fig 2). The meta-analysis of the MDAD and ITAAD subgroups meta-analysis demonstrated no significant difference in in-hospital mortality between the two groups ((OR 0.98; 95% CI 0.41–2.38, p = 0.970, I^2^ = 0%) and (OR 0.36; 95% CI 0.12–1.07, p = 0.070, I^2^ = 0%) respectively). A test for subgroup differences indicated no statistically significant difference between the two groups (p = 0.160) (Fig 2). Publication bias was assessed using a funnel plot (see supplementary information S2 Fig) along with regression-based Egger’s test (p = 0.498) and Begg’s test (p = 0.548), given the small number of studies, funnel plots and Egger/Begg tests have limited power; results are inconclusive regarding small-study effects. The leave-one-out sensitivity analysis validated that the results were robust and not substantially influenced by leaving out of a single study (see supplementary information S3 Fig).

#### Permanent neurological dysfunction and other post-operative complications.

**Fig 3:**
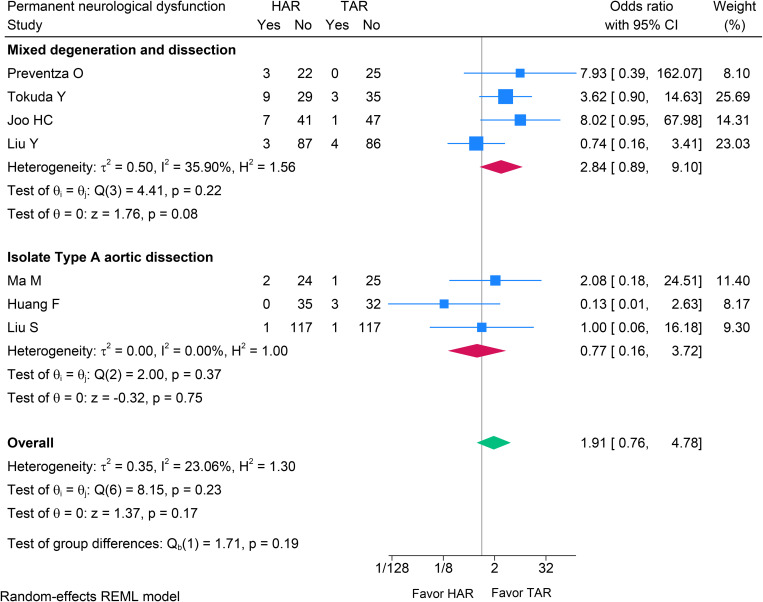
Forest plot showing the results of permanent neurological dysfunction after HAR versus TAR with the subgroup meta-analysis in ITAAD and MDAD aortic arch pathologies. PND: Permanent Neurological Dysfunction; HAR: Hybrid Arch Repair; TAR: Total Arch Replacement; ITAAD: Isolated Type A Aortic Dissection; MDAD: Mixed Degeneration and Dissection.

The incidence of permanent neurological dysfunction (PND) did not differ significantly between HAR and TAR group (OR 1.91; 95% CI 0.76–4.78; p = 0.170; I^2^ = 23.1%). Subgroup analyses likewise showed no statistically significant differences (p = 0.190) (see Fig 3), and overall heterogeneity was low. On the basis of PND risk alone, these data do not support a clinical preference for HAR over TAR; adequately powered studies are needed.

#### Renal failure, sternal re-entry due to hemorrhage and tracheostomy.

**Fig 4:**
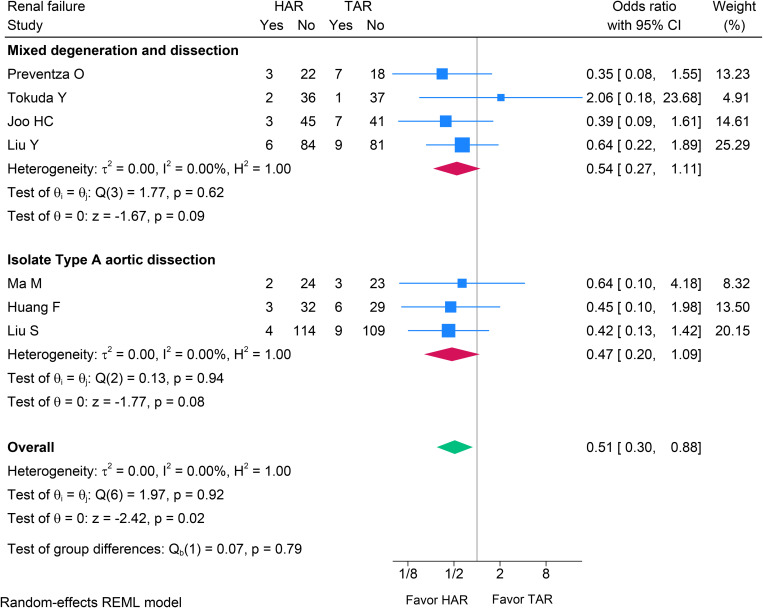
Forest plot shows the results of renal failure after HAR versus TAR. HAR: Hybrid Arch Repair; TAR: Total Arch Replacement.

Postoperative renal failure was evaluated across seven studies [21–23,29–32] and was significantly less frequent after HAR than after TAR (OR 0.51, 95% CI 0.30–0.88; p = 0.020) (see Fig 4), with no between-study heterogeneity (I^2^ = 0%). Subgroup meta-analyses produced pooled estimates favoring HAR but without statistical significance; the test for subgroup differences was also non-significant (p = 0.790). Although individual study CIs were wide and often crossed the null, the overall pooled estimate indicated a statistically significant reduction in renal failure with HAR and high consistency across studies.

**Fig 5:**
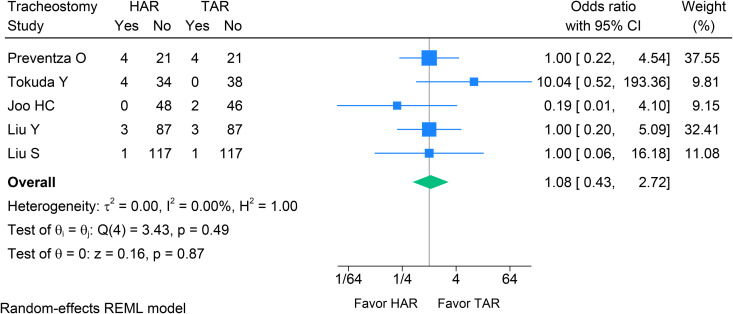
Forest plot shows the results of tracheostomy after HAR versus TAR. HAR: Hybrid Arch Repair; TAR: Total Arch Replacement.

**Fig 6:**
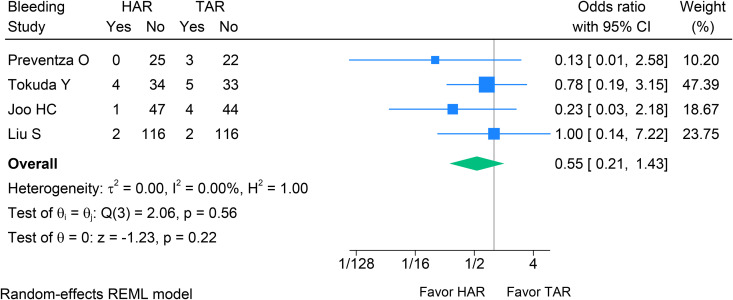
Forest plot shows the results of sternal re-entry due to bleeding after HAR versus TAR. HAR: Hybrid Arch Repair; TAR: Total Arch Replacement.

Across four studies [23,29,30,32], HAR showed no statistically significant difference in sternal re-entry for hemorrhage compared with TAR (OR 0.55, 95% CI 0.21–1.43, p = 0.220, I^2^ = 0%) (see Fig 4). In five studies [23,29–32], there was no evidence of a difference in tracheostomy between HAR and TAR (OR 1.08, 95% CI 0.43–2.72, p = 0.870, I^2^ = 0%; Fig 5). Therefore, bleeding was more favorable for HAR (OR 0.55, 95% CI 0.21–1.43, p = 0.220, I^2^ = 0%; Fig 6), while tracheostomy rates did not differ between the two groups.

### Intermediate-term survival rate

**Fig 7:**
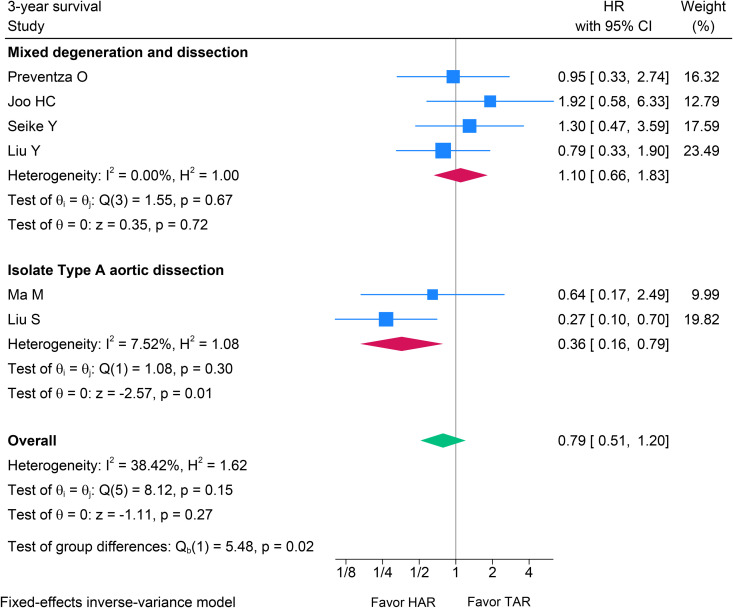
Forest plot illustrating the results of 3-year survival probability after HAR versus TAR with the subgroup meta-analysis in ITAAD and MDAD aortic arch pathologies. HAR: Hybrid Arch Repair; TAR: Total Arch Replacement; ITAAD: Isolated Type A Aortic Dissection; MDAD: Mixed Degeneration and Dissection.

Six studies were included [12,21,25–32], comprising 714 patients, with 357 pairs matched in each group. The aggregated results for the 3-year survival rate indicated no statistical difference between the HAR and TAR groups (HR 0.79; 95% CI 0.51–1.20, p = 0.270, I^2^ = 38.4%) (see Fig 7). Within the ITAAD subgroup, the 3-year survival probability was more advantageous for HAR (HR 0.36; 95% CI 0.16–0.79, p = 0.010, I^2^ = 7.52%). In the MDAD subgroup, no statistically significant difference in the hazard ratio was identified between the HAR and TAR groups (HR 1.10; 95% CI 0.66–1.83, p = 0.72, I^2^ = 0%) (see Fig 7). The analysis for subgroup differences indicates a statistically significant result (p = 0.020), suggesting that type A aortic dissection significantly alters the effect of HAR in comparison to TAR.

Only two studies [12,27,28,30] provided the 5-year survival outcome. The aggregated results indicated no statistically significant difference between the TAR and HAR groups (HR 1.54; 95% CI 0.77–3.11, p = 0.220; I^2^ = 0%) (see supporting information S4 Fig).

### Freedom from re-intervention probability

**Fig 8:**
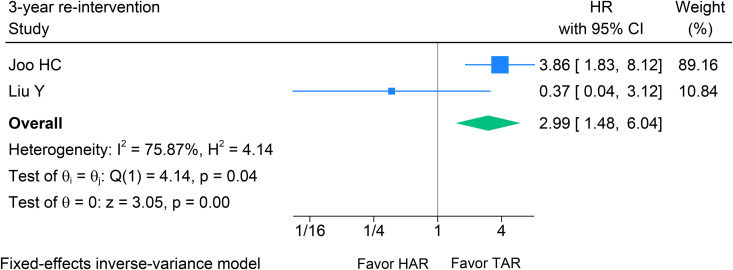
Forest plot shows the results of 3-year freedom from re-intervention after HAR versus TAR. HAR: Hybrid Arch Repair; TAR: Total Arch Replacement.

Only two studies [25,27,28,30,31] were analyzed, comprising 276 patients assigned to 138 matched pairs in each group. The overall findings for the 3-year freedom from re-intervention revealed a significantly higher rate of re-intervention in the HAR group compared to the TAR group (HR 2.99; 95% CI 1.48–6.04, p < 0.001; I^2^ = 75.9%) (see Fig 8). However, due to the small number of studies and high heterogeneity, these findings should be interpreted with caution.

## Discussion

The primary outcome of our analysis indicated no significant difference in in-hospital mortality across all studies (p = 0.240). The subgroup analysis indicated no statistically significant difference in overall in-hospital mortality compared to the subgroup of ITAAD studies (p = 0.160). Our results indicated a potential pattern suggesting a trend in in-hospital mortality advantage for ITAAD patients underwent HAR compared to TAR (OR 0.36; 95% CI 0.12–1.07, p = 0.07). This finding was consistent with prior studies [21,22,32], including individuals with ITAAD who underwent Type II hybrid debranching surgery, as proposed by Bavaria et al. [33]. The propensity-matched cohort studies indicated that in-hospital mortality varied from 1.7% and 7.7% in patients with HAR and from 2.5% to 23.1% in patients with TAR [21,22,32], regardless of whether they had undergone a frozen elephant trunk procedure. Our meta-analysis findings indicate that in-hospital mortality is comparable to the study reported by Bavaria et al. for Type I and Type II HAR in patients with aortic arch aneurysms [33].

Our analysis estimated the odds ratio of PND following HAR compared TAR. In studies of MDAD aortic pathologies, there was a trend toward higher stroke rates in the HAR group than in TAR group, but the difference was not statistically significant (OR 2.84; 95% CI 0.89–9.10; p = 0.080). This finding was consistent with a study from Iba et al. [25] and Hiroaka et al. [26], which similarly indicated a higher risk of stroke in the HAR. However, no significant difference was observed in the subgroup of study involving ITAAD. In contrast, Eleshra et al. [34] documented stroke rates of 14% in patients with degenerative aneurysms and 2% in patients with aortic dissection. Huang et al. [22] reported a lower incidence of stroke in HAR among patients with ITAAD, although these results had no statistically significant (OR 0.13; 95% CI 0.01–2.63).

Our analysis demonstrated a statistically significant reduction in the incidence of renal failure (OR 0.51; 95% CI 0.30–0.88; p = 0.020). These results corresponded with the findings of the majority of other studies [24,27,29,30]. A large non-propensity matched cohort study by Wallen et al. [35] reported lower rates of mortality, stroke, paralysis, and renal failure in the TAR group, highlighting the potential influence of selection bias, and the TAR group tended to enroll younger patients with fewer cases of peripheral arterial disease, and less preoperative hemodialysis.

We combined 3-year survival probability between HAR and TAR, revealing no statistically significant difference (HR 0.79; 95% CI 0.51–1.20). However, significant differences were observed between the overall outcome and the ITAAD subgroup. These meta-analyses indicated a trend suggesting that ITAAD may benefit from HAR. At this point, clinical application should be approached with caution: the subgroup meta-analysis included only two studies, lowering statistical power and increasing the probability of chance findings. Additional evidence is required to support this hypothesis.

The 3-year re-intervention rate was significantly higher in the HAR group (HR 2.99; 95% CI 1.48–6.04, p < 0.001), suggesting inferior re-intervention outcomes compared to TAR regarding freedom from re-intervention. In this study, statistical power was limited, and heterogeneity was high, but the findings suggest that HAR may carry a higher three-year re-intervention hazard than TAR. These results should inform shared decision-making: TAR may be preferred when operative risk is acceptable and long-term durability is the priority. Further research with methodologically robust designs and rigorous handling of selection bias is needed.

Recently, Spath et al. [36] published a meta-analysis that aggregated outcomes of aortic arch repairs utilizing endovascular techniques for chronic aortic dissections, degenerative aortic aneurysms, penetrating aortic ulcers, and aortic pseudoaneurysms. The overall technical success rate was 95.5% accompanied by 30-day mortality rate of 6.7%. The findings may be comparable to those in unmatched cohorts for the HAR group, which reported mortality rates ranging from 2.9% to 11.1% [22,24,27,29,30]. The study also emphasized that evidence on immediate through long-term outcomes after total endovascular repairs remains limited, raising questions about endograft durability.

Results from prior single-center study may be constrained by several factors, including sample size, surgeon experience, and surgical preference, all of which contribute to selection bias, a significant confounding factor. These factors may complicate the assessment of the therapeutic effects of HAR. Although bias can be mitigated by using propensity score matching [37], small studies may still lack the statistical power necessary to yield reliable results [38]. The data collected from this meta-analysis may be beneficial in assessing therapeutic efficacy of HAR compared to conventional TAR. The allocation of surgical procedures among individuals with various aortic arch pathologies or within specific subgroups may be informed by these results.

### Implication of this study

Our study is the first systematic review and meta-analysis aimed at estimating treatment outcomes between HAR and TAR, with a particular emphasis on mitigating selection bias through the inclusion of only propensity-score matched studies. The selection of surgical treatment for aortic arch pathologies remains crucial.

The findings suggest that HAR confers a significant advantage over TAR in reducing renal failure. In the type A aortic dissection subgroup, HAR also showed a trend toward lower in-hospital mortality and higher three-year survival. Conversely, in the mixed degeneration and dissection subgroup, TAR showed a non-significant trend toward lower rates of permanent neurological dysfunction and three-year re-intervention. These subgroup meta-analysis results should be interpreted with caution: the small number of studies and heterogeneity limit the robustness of the evidence.

### Limitation

A major limitation of this meta-analysis was the insufficient reporting of missing data across the analyzed retrospective cohorts. Therefore, this omission could cause biases, resulting in an inaccurate calculation of impact magnitude or creating a misleading impression of precision. According to the ROBINS-I tool, these issues warrant a “serious” categorization, indicating a potential (rather than established) risk of bias. Furthermore, the specific details in the characteristics of aortic aneurysm and the entry location of aortic dissection were absent in the included research, and hybrid arch debranching surgery for aortic dissection represents the sole study from China included in this meta-analysis. These details are essential for evaluating the relevance of the surgical outcomes to various patient subgroups and could impact the generalizability of our conclusions.

Long-term follow-up of surgical interventions, particularly those utilizing propensity score–matched cohorts, is essential. Long-term studies are required to understand the durability and long-term efficacy of various of these treatments. The apparently inconsistent outcomes between TAR and HAR in the subgroup meta-analysis may merely indicate inadequate statistical power due to limited number of studies included. Future research should concentrate on prospective observational studies, propensity score matching, or randomized controlled trials relevant to specific aortic arch pathologies. These methodologies are essential for obtaining less biased estimates of surgical effects and for validating our meta-analysis findings.

Finally, developing a patient-specific decision algorithm to guide procedures selection is crucial. Such an approach would allocate surgical strategies according to defined clinical characteristics and conditions, thereby maximizing patient benefit.

## Conclusions

This meta-analysis is the first study to systematically evaluate and compare the outcomes of HAR and TAR while utilizing propensity score-matched studies, specifically aimed at mitigating selection bias. The HAR was associated with a lower risk of renal failure than TAR. In the type A aortic dissection subgroup, HAR showed a trend toward lower in-hospital mortality and better three-year survival. In mixed degeneration-dissection subgroup, TAR showed a non-significant trend toward lower risk of permanent neurological dysfunction and fewer three-year re-interventions. These subgroup findings should be interpreted with caution given the small study numbers, heterogeneity, and predominantly observational designs, which leave estimates imprecise and vulnerable to residual confounding. Larger, contemporary, methodologically rigorous studies are needed; until then, procedure selection should be individualized, balancing patient risk, anatomical suitability, and center expertise.

## Supporting information

S1 Fig Risk of bias assessment using the ROBINS-I tool for included non-randomized studies. (TIF)

S2 Fig Funnel plot. (TIF)

S3 Fig Leave-one-out meta-analysis of in-hospital mortality. (TIF)

S4 Fig Forest plot showing the results of 5-year survival after HAR versus TAR. HAR: Hybrid Arch Repair; TAR: Total Arch Replacement.
(TIF)

## References

[1] Mestres CA, Greco E, Madrid CG, Pomar JL. The ability of Salmonella to drill holes in the aorta. Eur J Cardiothorac Surg. 2002;22(1):145. doi: 10.1016/s1010-7940(02)00223-3 12103390

[2] Koechlin L, Schuerpf J, Bremerich J, Sommer G, Gahl B, Reuthebuch O, et al. Acute aortic dissection with entry tear at the aortic arch: long-term outcome. Interact Cardiovasc Thorac Surg. 2021;32(1):89–96. doi: 10.1093/icvts/ivaa228 33221851 PMC8906687

[3] Okada K. Total arch replacement: When and how?. Asian Cardiovasc Thorac Ann. 2023;31(1):42–7. doi: 10.1177/02184923211073374 35509182

[4] Ram E, Lau C, Dimagli A, Chu N-Q, Soletti G Jr, Gaudino M, et al. Reoperative total arch replacement after previous cardiovascular surgery: Outcomes in 426 consecutive patients. J Thorac Cardiovasc Surg. 2024;168(4):963-972.e2. doi: 10.1016/j.jtcvs.2023.08.035 37657714

[5] Tsagakis K, Pacini D, Grabenwöger M, Borger MA, Goebel N, Hemmer W, et al. Results of frozen elephant trunk from the international E-vita Open registry. Ann Cardiothorac Surg. 2020;9(3):178–88. doi: 10.21037/acs-2020-fet-25 32551250 PMC7298229

[6] Shrestha M, Martens A, Kaufeld T, Beckmann E, Bertele S, Krueger H, et al. Single-centre experience with the frozen elephant trunk technique in 251 patients over 15 years. Eur J Cardiothorac Surg. 2017;52(5):858–66. doi: 10.1093/ejcts/ezx218 28977379

[7] Czerny M, Grabenwöger M, Berger T, Aboyans V, Della Corte A, Chen EP, et al. EACTS/STS Guidelines for diagnosing and treating acute and chronic syndromes of the aortic organ. Eur J Cardiothorac Surg. 2024;65(2):ezad426. doi: 10.1093/ejcts/ezad426 38408364

[8] Rong D, Zhang H, Guo W. Aortic arch aneurysm isolated by percutaneous total endovascular arch replacement. Eur Heart J. 2022;43(30):2905. doi: 10.1093/eurheartj/ehac326 35706411 PMC9356906

[9] Gouveia E Melo R, Stana J, Prendes CF, Kölbel T, Peterss S, Stavroulakis K, et al. Current state and future directions of endovascular ascending and arch repairs: The motion towards an endovascular Bentall procedure. Semin Vasc Surg. 2022;35(3):350–63. doi: 10.1053/j.semvascsurg.2022.07.001 36153076

[10] Castrovinci S, Pacini D, Di Marco L, Berretta P, Cefarelli M, Murana G, et al. Surgical management of aortic root in type A acute aortic dissection: a propensity-score analysis. Eur J Cardiothorac Surg. 2016;50(2):223–9. doi: 10.1093/ejcts/ezw038 26941248

[11] Beckmann E, Martens A, Kaufeld T, Natanov R, Krueger H, Haverich A, et al. Is total aortic arch replacement with the frozen elephant trunk procedure reasonable in elderly patients?. Eur J Cardiothorac Surg. 2021;60(1):131–7. doi: 10.1093/ejcts/ezab063 33582774

[12] Seike Y, Matsuda H, Fukuda T, Hori Y, Inoue Y, Omura A, et al. Is debranching thoracic endovascular aortic repair acceptable as the first choice for arch aneurysm in the elderly?. Interact Cardiovasc Thorac Surg. 2019;29(1):101–8. doi: 10.1093/icvts/ivz027 30805619

[13] Page MJ, McKenzie JE, Bossuyt PM, Boutron I, Hoffmann TC, Mulrow CD, et al. The PRISMA 2020 statement: an updated guideline for reporting systematic reviews. BMJ. 2021;372:n71. doi: 10.1136/bmj.n71 33782057 PMC8005924

[14] Richardson WS, Wilson MC, Nishikawa J, Hayward RS. The well-built clinical question: a key to evidence-based decisions. ACP J Club. 1995;123(3):A12-3. doi: 10.7326/acpjc-1995-123-3-a12 7582737

[15] Higgins JPT, Thomas J, Chandler J, Cumpston M, Li T, Page MJ. Cochrane Handbook for Systematic Reviews of Interventions version 6.4 (updated August 2023). Cochrane. 2023.

[16] Sterne JA, Hernán MA, Reeves BC, Savović J, Berkman ND, Viswanathan M, et al. ROBINS-I: a tool for assessing risk of bias in non-randomised studies of interventions. BMJ. 2016;355:i4919. doi: 10.1136/bmj.i4919 27733354 PMC5062054

[17] Hozo SP, Djulbegovic B, Hozo I. Estimating the mean and variance from the median, range, and the size of a sample. BMC Med Res Methodol. 2005;5:13. doi: 10.1186/1471-2288-5-13 15840177 PMC1097734

[18] Parmar MK, Torri V, Stewart L. Extracting summary statistics to perform meta-analyses of the published literature for survival endpoints. Stat Med. 1998;17(24):2815–34. doi: 10.1002/(sici)1097-0258(19981230)17:24<2815::aid-sim110>3.0.co;2-8 9921604

[19] Williamson PR, Smith CT, Hutton JL, Marson AG. Aggregate data meta-analysis with time-to-event outcomes. Stat Med. 2002;21(22):3337–51. doi: 10.1002/sim.1303 12407676

[20] Tierney JF, Stewart LA, Ghersi D, Burdett S, Sydes MR. Practical methods for incorporating summary time-to-event data into meta-analysis. Trials. 2007;8:16. doi: 10.1186/1745-6215-8-16 17555582 PMC1920534

[21] Ma M, Feng X, Wang J, Dong Y, Chen T, Liu L, et al. Acute Type I aortic dissection: a propensity-matched comparison of elephant trunk and arch debranching repairs. Interact Cardiovasc Thorac Surg. 2018;26(2):183–9. doi: 10.1093/icvts/ivx283 29049664

[22] Huang F, Li X, Zhang Z, Li C, Ren F. Comparison of two surgical approaches for acute type A aortic dissection: hybrid debranching versus total arch replacement. J Cardiothorac Surg. 2022;17(1):166. doi: 10.1186/s13019-022-01920-9 35739545 PMC9229500

[23] Tokuda Y, Oshima H, Narita Y, Abe T, Araki Y, Mutsuga M, et al. Hybrid versus open repair of aortic arch aneurysms: comparison of postoperative and mid-term outcomes with a propensity score-matching analysis. Eur J Cardiothorac Surg. 2016;49(1):149–56. doi: 10.1093/ejcts/ezv063 25732968

[24] Oishi Y, Kumamaru H, Kato M, Ohki T, Shiose A, Motomura N, et al. Open Versus Zone 0/1 Endovascular Aortic Repair for Arch Aneurysm: A Propensity Score-Matched Study from the National Clinical Database in Japan. Ann Vasc Surg. 2024;100:128–37. doi: 10.1016/j.avsg.2023.10.012 38122978

[25] Iba Y, Minatoya K, Matsuda H, Sasaki H, Tanaka H, Oda T, et al. How should aortic arch aneurysms be treated in the endovascular aortic repair era? A risk-adjusted comparison between open and hybrid arch repair using propensity score-matching analysis. Eur J Cardiothorac Surg. 2014;46(1):32–9. doi: 10.1093/ejcts/ezt615 24431168

[26] Hiraoka A, Chikazawa G, Totsugawa T, Tamura K, Ishida A, Sakaguchi T, et al. Objective analysis of midterm outcomes of conventional and hybrid aortic arch repair by propensity-score matching. J Thorac Cardiovasc Surg. 2017;154(1):100-106.e1. doi: 10.1016/j.jtcvs.2016.12.060 28314530

[27] Hori D, Okamura H, Yamamoto T, Nishi S, Yuri K, Kimura N, et al. Early and mid-term outcomes of endovascular and open surgical repair of non-dissected aortic arch aneurysm†. Interact Cardiovasc Thorac Surg. 2017;24(6):944–50. doi: 10.1093/icvts/ivx031 28329032

[28] Yoshitake A, Okamoto K, Yamazaki M, Kimura N, Hirano A, Iida Y, et al. Comparison of aortic arch repair using the endovascular technique, total arch replacement and staged surgery†. Eur J Cardiothorac Surg. 2017;51(6):1142–8. doi: 10.1093/ejcts/ezx028 28329146

[29] Preventza O, Garcia A, Cooley DA, Haywood-Watson RJL, Simpson K, Bakaeen FG, et al. Total aortic arch replacement: A comparative study of zone 0 hybrid arch exclusion versus traditional open repair. J Thorac Cardiovasc Surg. 2015;150(6):1591–8; discussion 1598-600. doi: 10.1016/j.jtcvs.2015.08.117 26573355

[30] Joo H-C, Youn Y-N, Kim J-H, Lee SH, Lee S, Yoo K-J. Conventional Open Versus Hybrid Arch Repair of Aortic Arch Disease: Early and Long-Term Outcomes. Ann Thorac Surg. 2019;107(5):1380–8. doi: 10.1016/j.athoracsur.2018.10.050 30508531

[31] Liu Y, Liang S, Zhang B, Dun Y, Guo H, Qian X, et al. Type II hybrid arch repair versus total arch replacement with frozen elephant trunk: a propensity score-matched analysis. Eur J Cardiothorac Surg. 2021;60(2):297–304. doi: 10.1093/ejcts/ezab047 33939801

[32] Liu S, Qiu J, Qiu J, Jiang W, Gao W, Wei B, et al. Midterm Outcomes of One-Stage Hybrid Aortic Arch Repair for Stanford Type A Aortic Dissection: A Single Center’s Experience. Semin Thorac Cardiovasc Surg. 2023;35(2):311–21. doi: 10.1053/j.semtcvs.2021.12.016 35276357

[33] Bavaria J, Vallabhajosyula P, Moeller P, Szeto W, Desai N, Pochettino A. Hybrid approaches in the treatment of aortic arch aneurysms: postoperative and midterm outcomes. J Thorac Cardiovasc Surg. 2013;145(3 Suppl):S85-90. doi: 10.1016/j.jtcvs.2012.11.044 23260461

[34] Eleshra A, Heo W, Lee K-H, Kim T-H, Sim SA, Sharafeldin H, et al. Mid-term outcomes of hybrid debranching endovascular aortic arch repair in landing zones 0-2. Vascular. 2023;31(3):447–54. doi: 10.1177/17085381211068230 35100906

[35] Wallen T, Carter T, Habertheuer A, Badhwar V, Jacobs JP, Yerokun B, et al. National Outcomes of Elective Hybrid Arch Debranching with Endograft Exclusion versus Total Arch Replacement Procedures: Analysis of the Society of Thoracic Surgeons Adult Cardiac Surgery Database. Aorta (Stamford). 2021;9(1):21–9. doi: 10.1055/s-0041-1724003 34607380 PMC8489998

[36] Spath P, Campana F, Tsilimparis N, Gallitto E, Pini R, Faggioli G, et al. Outcomes of Fenestrated and Branched Endografts for Partial and Total Endovascular Repair of the Aortic Arch - A Systematic Review and Meta-Analysis. Eur J Vasc Endovasc Surg. 2024;67(1):106–16. doi: 10.1016/j.ejvs.2023.07.048 37536517

[37] D’Agostino RB Jr. Propensity score methods for bias reduction in the comparison of a treatment to a non-randomized control group. Stat Med. 1998;17(19):2265–81. doi: 10.1002/(sici)1097-0258(19981015)17:19<2265::aid-sim918>3.0.co;2-b 9802183

[38] Austin PC. An Introduction to Propensity Score Methods for Reducing the Effects of Confounding in Observational Studies. Multivariate Behav Res. 2011;46(3):399–424. doi: 10.1080/00273171.2011.568786 21818162 PMC3144483

