# Supplementary figures and images for: Comparison of early and intermediate-term outcomes between hybrid arch debranching and total arch replacement: A systematic review and meta-analysis of propensity-matched studies
Source: PLoS One. 2025 Sep 4;20(9):e0314341. doi: 10.1371/journal.pone.0314341 (PMC12410729; doi:10.1371/journal.pone.0314341)

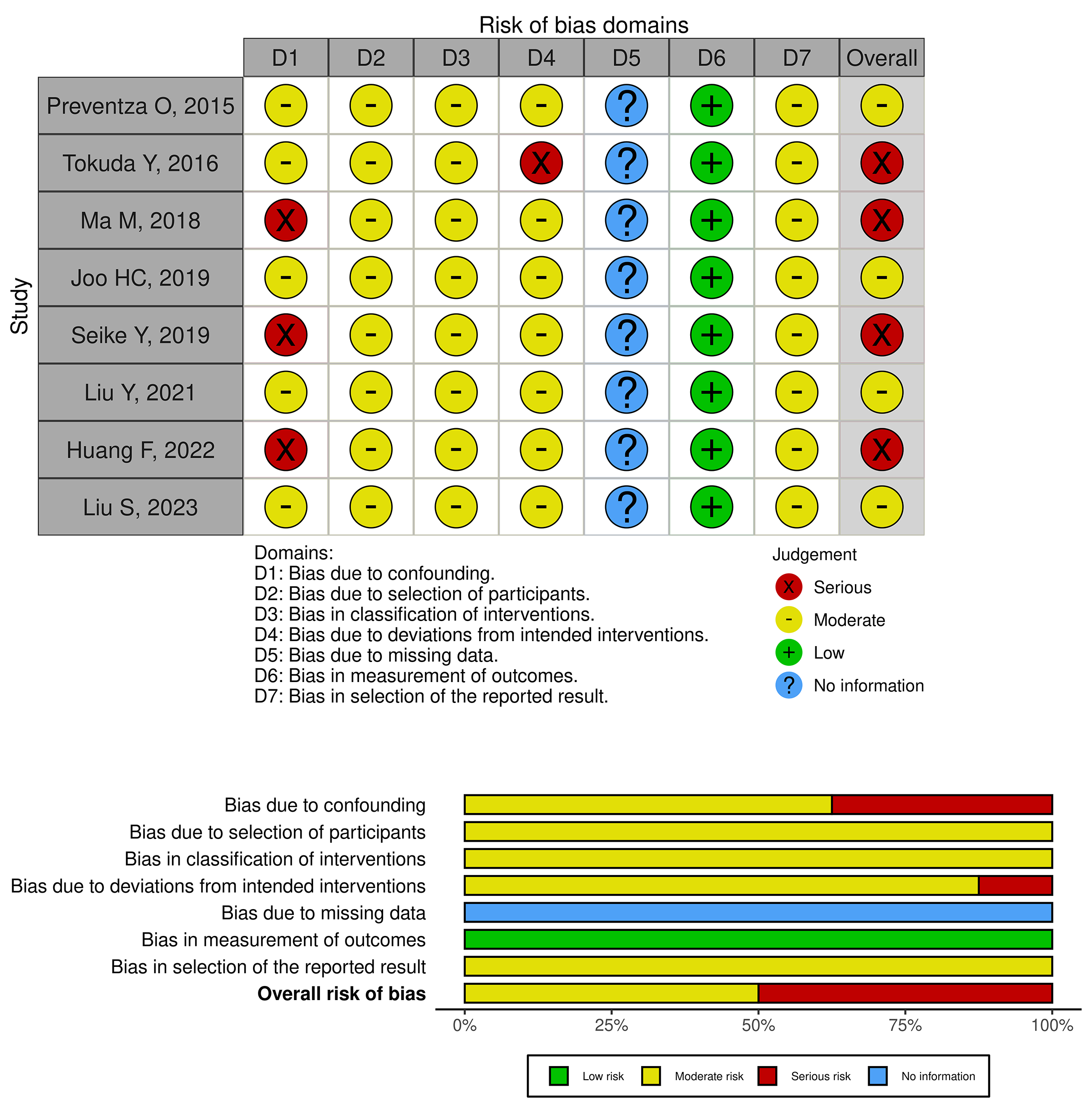

Supplement: S1 Fig — (TIF) [file pone.0314341.s001.tif]

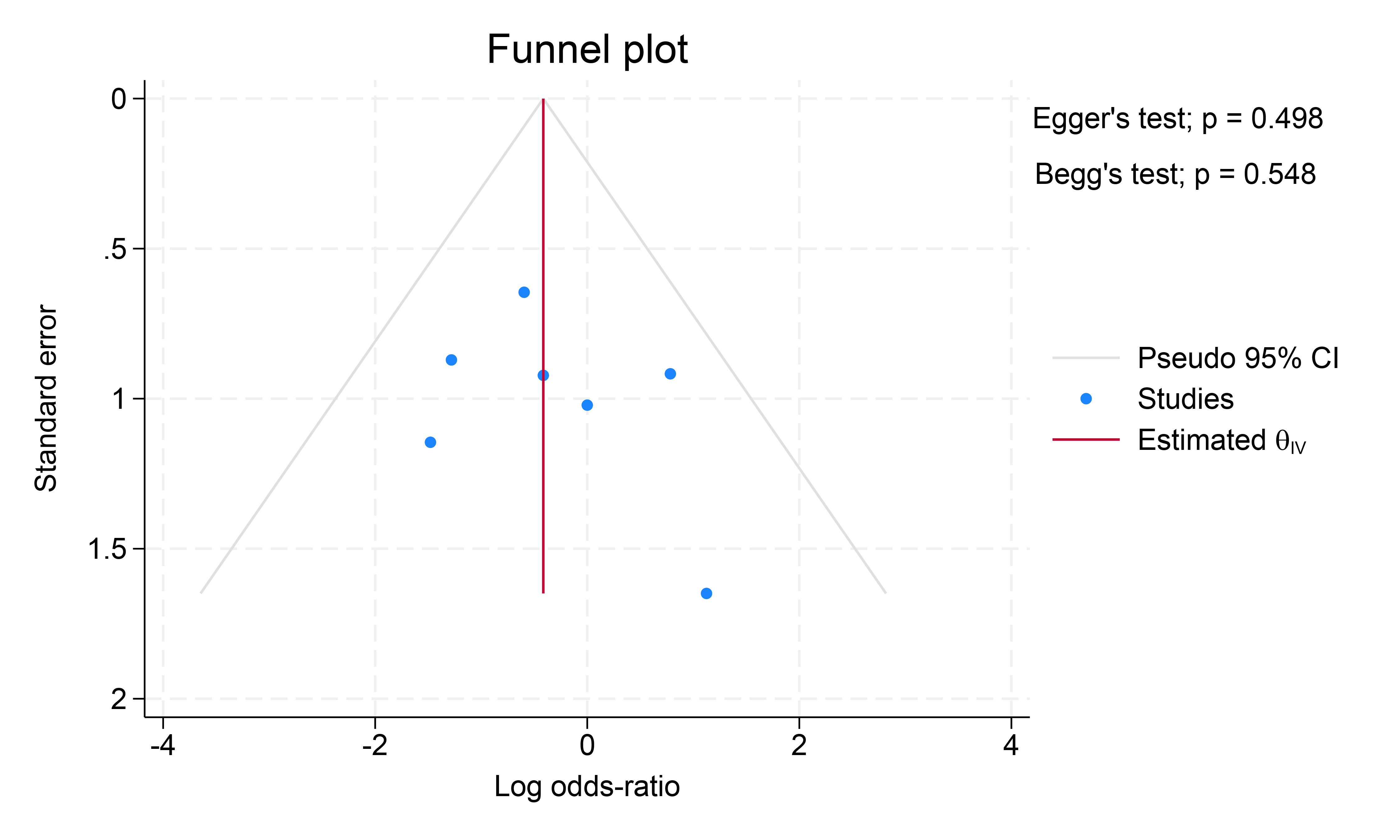

Supplement: S2 Fig — (TIF) [file pone.0314341.s002.tif]

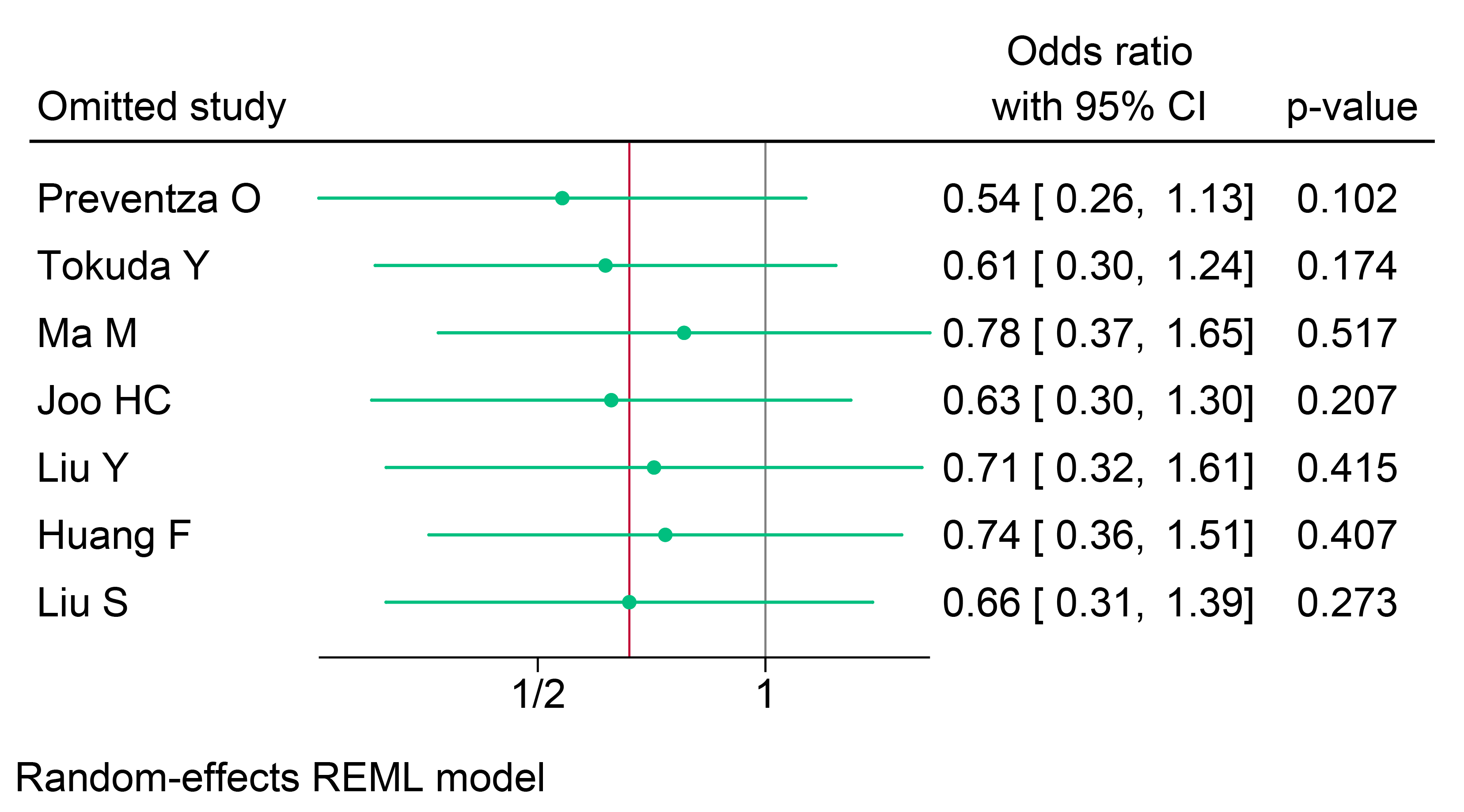

Supplement: S3 Fig — (TIF) [file pone.0314341.s003.tif]

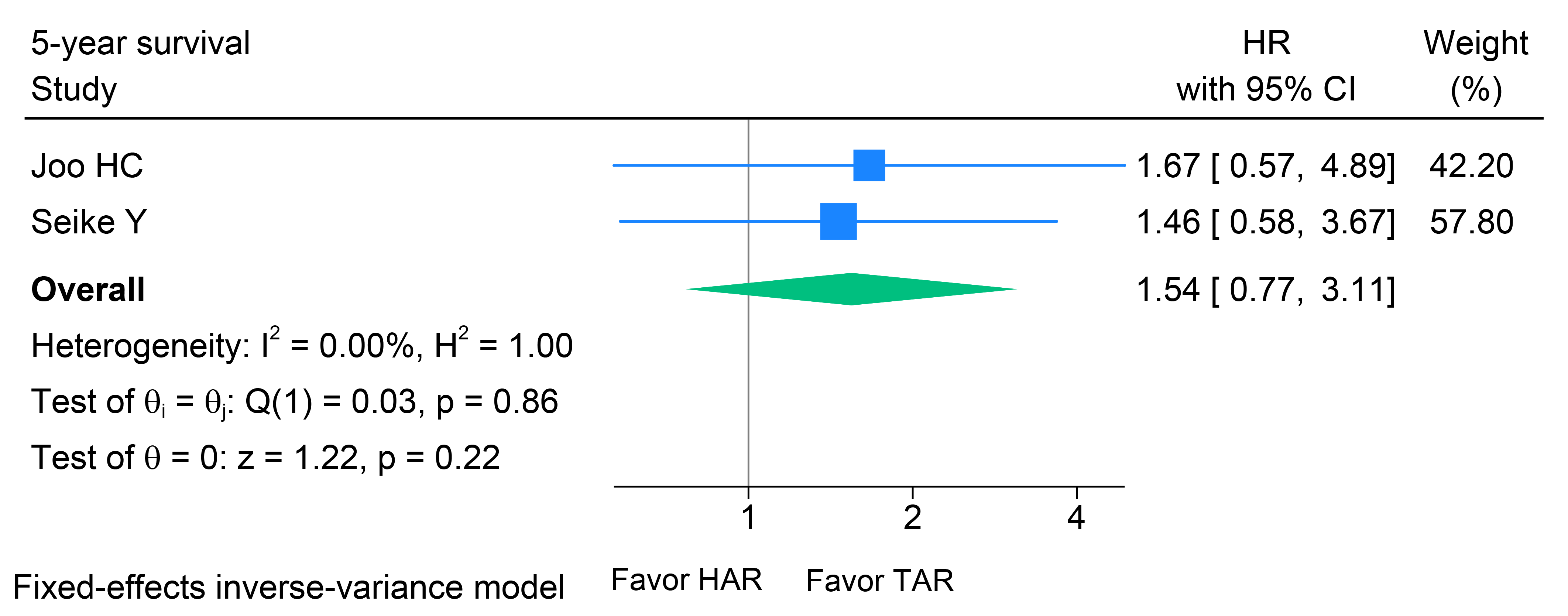

Supplement: S4 Fig — HAR: Hybrid Arch Repair; TAR: Total Arch Replacement. (TIF) [file pone.0314341.s004.tif]
